# Traditional African remedies induce hemolysis in a glucose-6-phopshate dehydrogenase deficient zebrafish model

**DOI:** 10.1038/s41598-020-75823-x

**Published:** 2020-11-05

**Authors:** Olufunmilayo Arogbokun, Margaret Shevik, Tina Slusher, Zubaida Farouk, Alexis Elfstrum, Jenna Weber, Sarah E. Cusick, Troy Lund

**Affiliations:** 1 The University of North Carolina at Chapel Hill, Chapel Hill, NC USA; 2 Pediatric Blood and Marrow Transplant Program, Global Pediatrics, University of Minnesota, MMC 366, 420 Delaware St SE, Minneapolis, MN 55455 USA; 3 Department of Pediatrics, Hennepin Healthcare University of Minnesota, Minneapolis, USA; 4 Bowen University of Teaching Hospital, Ogbomoso, Nigeria; 5 Department of Paediatrics, Aminu Kano Teaching Hospital, Bayero University, Kano, Nigeria; 6 Utah School of Medicine, Salt Lake City, UT USA; 7 Division of Epidemiology, Department of Pediatrics, University of Minnesota, Minneapolis, USA

**Keywords:** Haematological diseases, Neurological disorders, Nutrition disorders

## Abstract

Traditional remedies are widely used throughout Africa in routine care for infants. However, such remedies could have detrimental effects. Acute bilirubin encephalopathy (ABE) and kernicterus spectrum disorder (KSD) are common newborn health conditions in the developing world, contributing to substantial neonatal mortality and morbidity. They frequently occur in children with glucose-6-phopshate dehydrogenase (G6PD) deficiency. Using our established zebrafish model of G6PD deficiency, we tested the effects of three traditional compounds used in the care of the newborn umbilical cord: eucalyptus oil, methylated spirits, and Yoruba herbal tea. We found that eucalyptus oil induced a 13.4% increase in a hemolytic phenotype versus control, while methylated spirits showed a 39.7% increase in affected phenotype. Yoruba herbal tea exposure showed no effect. While methylated spirits are already a known pro-oxidant, these data indicate that eucalyptus oil may also be a hemolytic trigger in those with G6PD deficiency. Discovering which agents may contribute to the pathophysiology of G6PD deficiency is critical to eliminate ABE and KSD, especially in countries with a high prevalence of G6PD deficiency. The next step in elucidating the role of these agents is to determine the clinical correlation between the use of these agents and ABE/KSD.

## Introduction

The World Health Organization estimates that up to 80% of Africa uses traditional medicine for primary health care^1^, but the efficacy and safety of these treatments has not yet been scientifically established. Within Nigeria, traditional remedies are used prominently^2^. The Roll Back Malaria Partnership recently found that the first choice of treatment for high fever in Nigerian children is an herbal treatment^3^. Other studies have shown that mothers commonly use these traditional remedies for their neonates, both for treatment and for routine care^1,4^. The remedies used are wide-ranging and include herbs, spices, and other local vegetative species.

Glucose-6-phosphate dehydrogenase (G6PD) deficiency is the most common X-linked genetic disease in the world with up to 400 million individuals affected. G6PD is the first step in the pentose phosphate shunt to product the important reducing unit NADPH. NADPH provides cellular protection against oxidative stress during cellular metabolism and also from external pro-oxidant exposure. While most cells have redundant cellular mechanisms to deal with oxidative stress, red blood cells rely solely on NADPH produced by G6PD, as they have no mitochondria nor nucleus. G6PD deficiency offers some protection against several malarial infections and is found in up to 30% of Africa populations depending on the region^5–7^. Unfortunately, many of the drugs used to treat malaria are strong pro-oxidants—with primaquine being the prototypical example—and cause drug-induced hemolysis in affected individuals.

African neonates have immature antioxidant cellular metabolism and are at increased risk of hyperbilirubinemia and kernicterus with and without acute hemolysis. Tight associations have been demonstrated between G6PD deficiency and jaundiced African neonates^8–12^.

Some traditional remedies such as mentholated rub, camphor, naphthalene, and henna have been studied and shown to cause hemolysis in neonates with G6PD deficiency^13–15^. We have observed the use of eucalyptus oil, methylated spirits, and traditional Yoruba tea for neonatal umbilical cord care as well as for general “healing properties.” Many of these infants going on to develop jaundice. With Nigeria having a high burden of G6PD deficiency^16^, large traditional remedy usage, and the recent prioritization of identification of effective treatments of neonatal jaundice by Nigerian pediatricians, it is important to explore the safety and impact of such treatments^12,17^.

We aimed to study the impacts of these widely used traditional remedies in our previously established G6PD-deficient zebrafish^18^. This model utilizes morpholino-induced downregulation of G6PD (at the 1–2 cell stage) followed by compound exposure. We have shown that this model produces a hemolytic anemia which generates a clear phenotype consisting of robust pericardial edema (and often anasarca, see Supplementary Figure 1). This system allowed rapid high throughput testing of compounds and has been shown to be very responsive to pro-oxidant exposures in producing the expected phenotype^18^.

## Results

**Figure 1:**
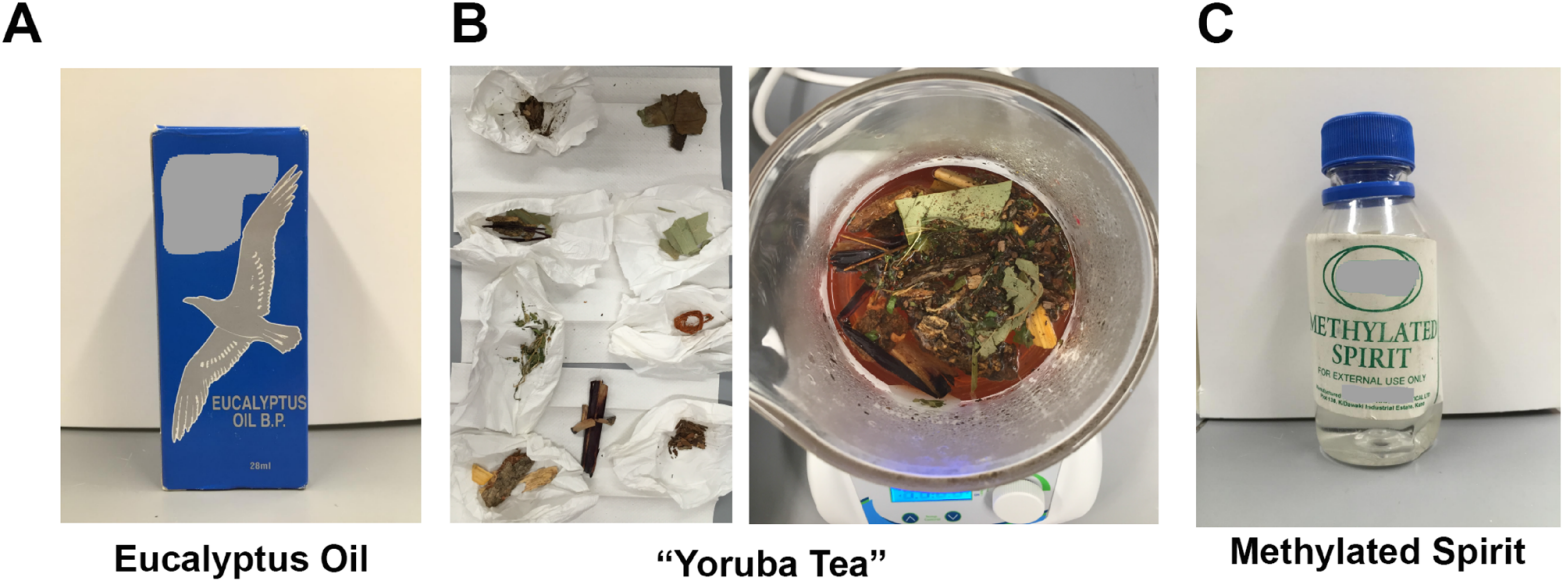
Compound tested in the G6PD-deficient zebrafish model. (**A**) Eucalyptus oil. (**B**) A variety of roots, bark, herbs to brew Yoruba tea (right panel). (**C**) Methylated spirits.

To investigate oxidative stress induced hemolysis, we tested eucalyptus oil, methylated spirit, and Yoruba tea in our G6PD-deficient zebrafish model^18^ (Fig. 1). Initial experiments to establish appropriate dilutions which did not cause high levels of toxicity or massive death were performed in wildtype animals.

**Figure 2:**
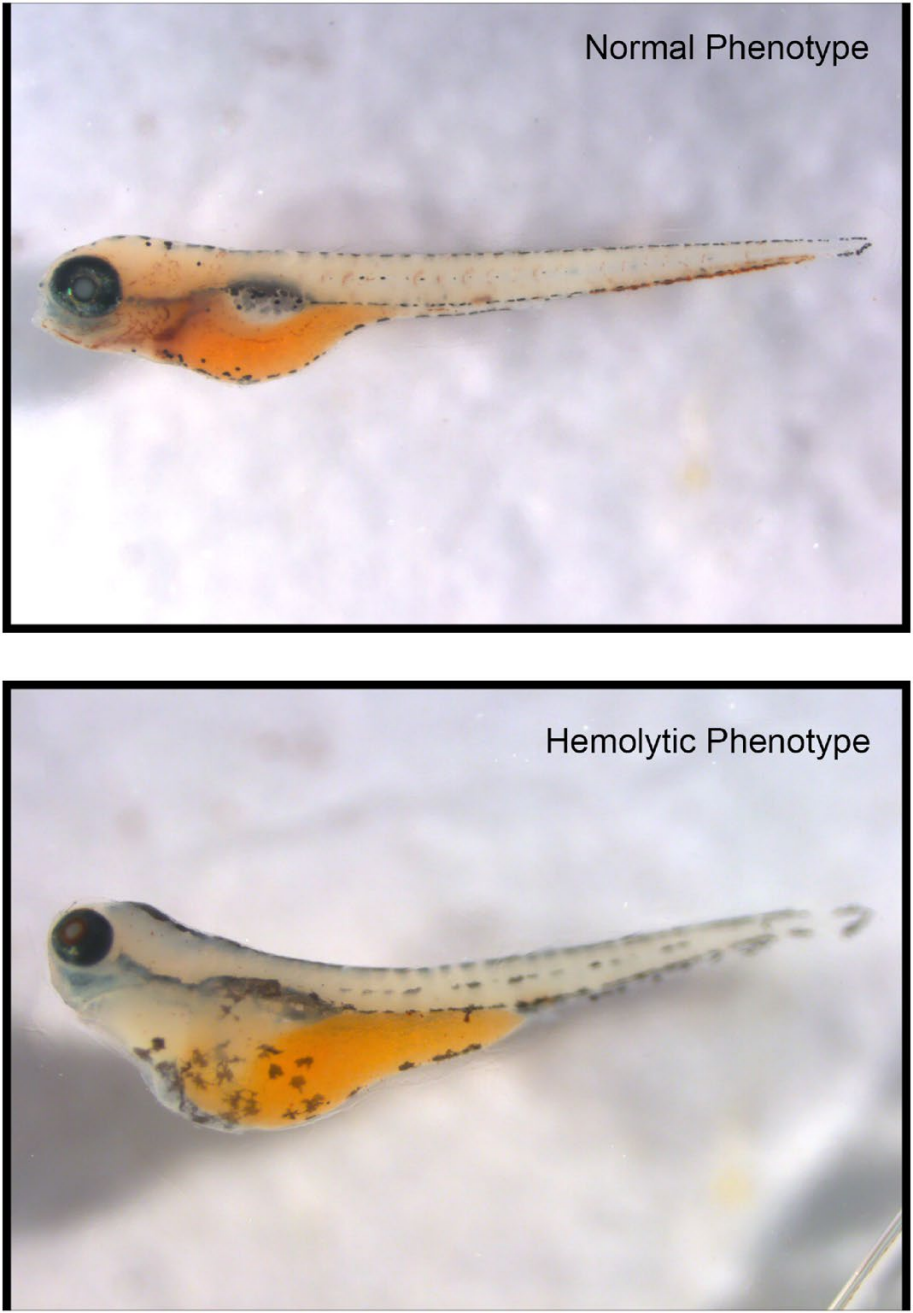
Hemolysis in a G6PD-deficient zebrafish model. Top micrograph shows a zebrafish at 72 hpf with intact red blood cells. Bottom micrograph shows an edematous G6PD-deficient zebrafish at 72 hpf after 48 h of methylated spirit exposure. Both animals were subject to o-dianisidine staining to show hemoglobin containing red cells. Zebrafish were imaged using a Leica DFC340FX fluorescent microscope with PlanAPO 1.6×/0.05 NA objective. Image capture was performed with Leica Application Suite X 3.6.0.2010 (https://www.leica-microsystems.com/products/microscope-software/p/leica-application-suite/).

G6PD deficiency was induced through the use of morpholino (MO) injections at the 1–2 cell stage as previously described^18^. At 24 h post-fertilization (hpf), embryos were dechorionated and exposure to selected compounds. After 48 h of compound exposure, animals were tallied for those with an edematous phenotype and those with a wild-type appearance (Fig. 2). Comparison of animals from uninjected, G6PD MO, and Random MO injected groups showed a low-level of hemolysis from injection alone, a known possible phenomenon when injection of foreign nucleic acid induces a stress response^18^.

**Figure 3:**
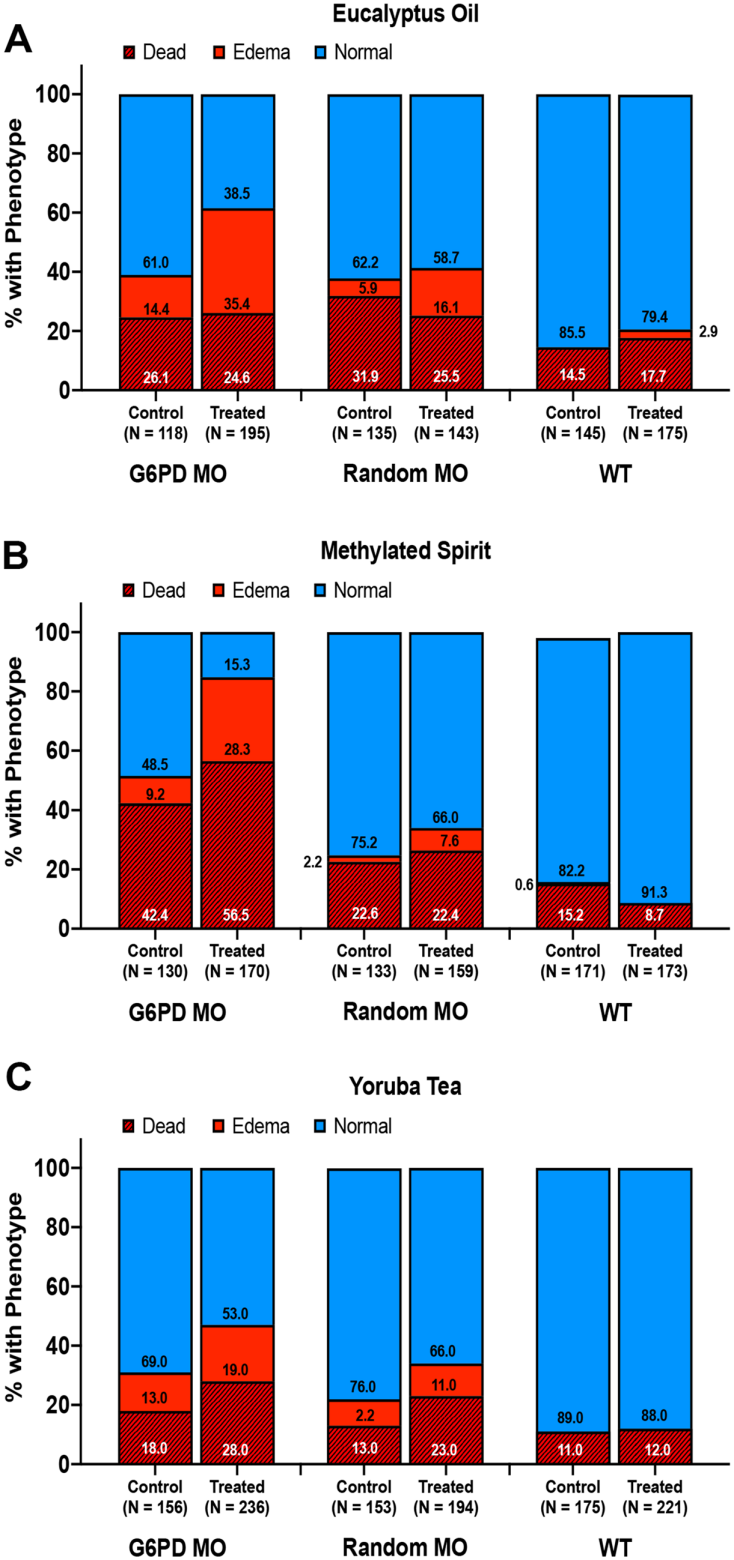
Enumeration of the hemolytic phenotype in G6PD-deficient zebrafish after compound exposure. (**A**) Impact of eucalyptus oil exposure and regular fishwater on zebrafish in the following groups: G6PD MO, and random MO, uninjected control group. (**B**) Impact of methylated spirits exposure versus regular fishwater on zebrafish in the following groups: G6PD MO, and random MO, uninjected control group. (**C**) Impact of Yoruba tea exposure versus regular fishwater on zebrafish in the following groups: G6PD MO, and random MO, uninjected control group.

We first tested eucalyptus oil diluted in fishwater and showed a significant loss of normal phenotype in G6PD morphants exposed to eucalyptus compared to non-exposure phenotype (38.5% normal versus 61.0% normal, respectively, Fig. 3A). As with our prior report, death is most often accompanied by a hemolytic phenotype^18^. Consequently, 61.5% of the G6PD morphants exposed to eucalyptus displayed hemolytic/dead phenotype compared to non-exposure animals at 39.0% (Fig. 3A).

We next tested methylated spirits and showed a significant loss of normal phenotype in G6PD morphants exposed to methylated spirits compared to non-exposure phenotype (15.3% normal versus 48.5% normal, respectively, Fig. 3B). Consequently, 84.7% of the G6PD morphants exposed to methylated spirits displayed hemolytic/dead phenotype compared to non-exposure animals at 51.5% (Fig. 3B). It should be noted that a clutch can experience a greater level of death at baseline, as these animals are very outbred, frequently giving rise to heterogeneity in offspring. In this clutch, the G6PD MO death rate was 42.3%.

Finally, Yoruba tea was made by boiling local ingredients for 20 min in fishwater. The tea was then diluted in fresh fishwater. The animals in the Yoruba tea environment showed some loss of normal phenotype in G6PD morphants compared to non-exposure phenotype (69% normal versus 53% normal, respectively, Fig. 3C). Consequently, 47% of the G6PD morphants exposed to Yoruba tea displayed hemolytic/dead phenotype compared to non-exposure animals at 31% (Fig. 3C).

**Figure 4:**
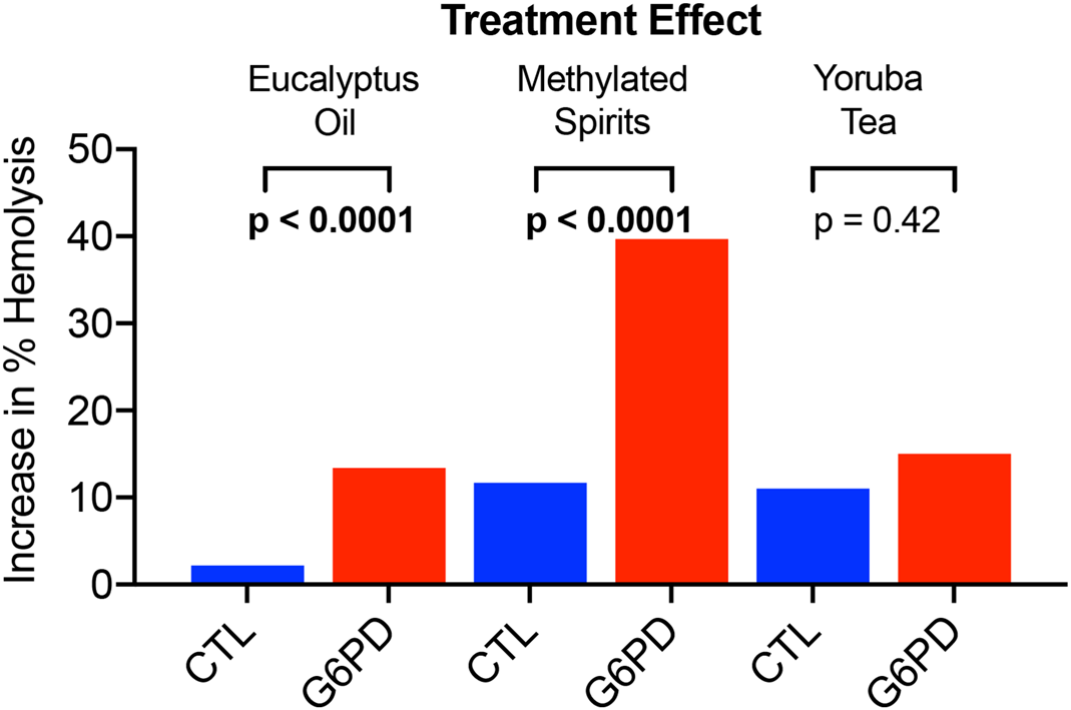
Treatment effect of compounds on G6PD-deficient zebrafish. Tallies were normalized to phenotype data produced in the unexposed embryos to produce a treatment effect. Random MO and G6PD MO injected animals were compared using multivariate analysis was used to separate effects from morpholino exposure versus compound exposure.

Although the data show a propensity for increased hemolysis in the eucalyptus and methylated spirit exposed G6PD morphants, there is always clutch-to-clutch variation and background phenotype generated from both compound exposure and MO injection. Therefore, in the unexposed morphants, we normalized by phenotype to produce a treatment effect and compared Random MO and G6PD MO injected animals. Figure 4 shows that eucalyptus oil produced a 13.4% increase in hemolysis in G6PD-deficient animals compared to a 2.2% increase in control animals (Random MO, p < 0.0001). Methylated spirit exposure produced a 39.7% increase in hemolysis in G6PD-deficient animals compared to 11.7% increase in control animals (Random MO, p < 0.0001). In contrast, Yoruba tea exposure produced a 11.0% increase in hemolytic phenotype in Random morphants and a 15.0% increase in G6PD morphants, though the difference was not statistically significant (p = 0.42).

## Discussion

Experimental results from these experiments indicate that eucalyptus and methylated spirits can trigger hemolytic anemia in the G6PD-deficient model. The use of both of these compounds are commonplace in newborn umbilical cord care, where absorption of the compound is likely. It may be unwise to use these remedies in the newborn given the high rates of G6PD deficiency in Nigeria.

Numerous agents have been shown to cause hemolysis in individuals with G6PD deficiency. Some, such as fava beans, henna, naphthalene, primaquine, dapsone, have conclusively been shown to cause hemolysis and thus should be avoided in individuals with G6PD deficiency^1,13,19–21^. However, in low-resource settings like Nigeria, most individuals do not know their G6PD status. Despite the long-standing recommendation that G6PD screening be done in any country with a G6PD prevalence of more than 3–5% of males, it is not routinely done in most low- and middle-income countries. Therefore, problematic or potentially problematic agents should be be avoided in all neonates in these countries^22^, and safer treatments should be a promoted.

Parents and clinicians have questioned whether the use of eucalyptus oil and herbs are dangerous in neonates with G6PD deficiency, but little literature exists to answer this important question. Multiple in vitro and in vivo studies suggest that eucalyptus has both antioxidant/anti-inflammatory and pro-oxidant activities. These opposite effects may be due to extraction method, plant source (*Eucalyptus* is a genus of many species), and the target organ being studied.

However, since severe neonatal hyperbilirubinemia is common in Africa and one of the primary causes is G6PD deficiency, it is crucial to determine which commonly used remedies should be avoided. In our study, zebrafish with G6PD deficiency exposed to eucalyptus oil treatment showed that the treatment triggers hemolytic anemia. These pre-clinical studies this suggest that eucalyptus oil may pose a danger to neonates with G6PD deficiency, but this potential threat must be validated in clinical studies.

Despite the current recommendation by the World Health Organization and others to transition to chlorohexidine for cord care, expense and availability have made this transition slow^23^. As noted in the study by Farouk et al., methylated spirits are also commonly used on the umbilical cords of neonates as a drying agent^19^. Spirits have been presumed to be safer than other native treatments such as plant material because of the risk of neonatal tetanus. However, methylated spirits have not been previously considered by most clinicians to be a risk factor for hemolysis in G6PD-deficient neonates, despite these spirits being a denatured alcohol (ethanol) and a well-described pro-oxidant in several organ systems, including the brain, heart, and liver^24–26^. As with eucalyptus oil, the results from zebrafish with G6PD deficiency exposed to methylated spirits also show a higher rate of hemolysis, suggesting, like eucalyptus, these spirits may be a concern for neonates with G6PD deficiency.

In contrast, diluted Yoruba tea treatment did not trigger hemolytic anemia in the G6PD-deficient zebrafish. This tea is often produced by mothers for cleaning of the umbilical cord as well as for ingestion (by mother and baby). Consequently, it is possible that Yoruba tea treatment does not pose as much risk to neonates who are G6PD-deficient, though it is likely not effective as a cleaning agent. The results of this study are limited by the tea concoction which comes from an assortment of organic compounds that likely vary widely by region. The amounts, concentrations, and treatment of each ingredient are likely also different. We have approximated a “best guess” based on local knowledge by the investigators.

As clinicians and researchers attempt to eliminate acute bilirubin encephalopathy and kernicterus spectrum disorder worldwide, it is essential that agents that are possible triggers for hemolysis in G6PD-deficient neonates be studied both in the lab and in clinical settings. Given the close association of neonatal jaundice and G6PD deficiency throughout much of the world, the results of this study need to be further explored epidemiologically through clinical correlation of exposure histories in infants with G6PD deficiency and jaundice.

## Methods

### Zebrafish strains and fish husbandry

Zebrafish (AB strain) were maintained by the University of Minnesota Zebrafish Core Facility. All methods were carried out in accordance with relevant guidelines and regulations. All experimental protocols were according to standardized procedures and approved by the University of Minnesota’s International Animal Care and Use Committee. Wild type fish were obtained from Segrest Farms (Gibsonton, FL, USA) and bred in-house.

### Morpholinos

The G6PD antisense translation blocker and splice morpholino’s (MO) were designed and purchased from Gene Tools (Philomath, OR, USA) and used as previously described^18^. The MOs were dissolved in water at a stock concentration of 8 mg/mL. 3 nL volumes of MO were delivered via air pressure injection into one- to two-cell stage embryos. The G6PD MOs were combined in a 1:1 ratio for delivery of 1.2 pmol. Random MOs at 1.2 pmol were used for control injection. The G6PD translation blocking the MO sequence was 5′-gcgctcgctcgactgcccatcattt-3′; the splice-blocking MO sequence was 5′-ataataaaagacttaccgaagcgccc-3′. The random MO was created with a similar GC content and random bases used. Random MOs from two different batches were used. Wildtype (WT) embryos were kept from each clutch as a non-injected control, a signal of the clutch’s overall health before treatment.

### Prooxidant exposure

All zebrafish embryos were dechorionated at 24 h post-fertilization (hpf) and placed in 6- or 12-well plates for treatment, depending on the size of the clutch. All chemicals were dissolved in zebrafish water (“fishwater”, 0.006% Instant Ocean in MilliQ water) prior to treatment. For each chemical, dose curves were established to determine which concentrations yielded the best survival rate for uninjected embryos. Final dosages varied between chemicals. Silverbird Eucalyptus Oil from Bell’s Healthcare in the United Kingdom was dissolved at a ratio of 1:5000 in fishwater. Methylated Spirits from Aicon Pharmaceuticals LTD was dissolved at a ratio of 1:5000 in fishwater. A Yoruba tea concoction consisting of various roots, leaves, bark, and herbs obtained from a shaman in Nigeria and boiled in fishwater for 20 min, was dissolved at a ratio of 1:75 in fishwater. Fresh dilutions were made for each new clutch, with treatment lasting until 72 hpf as previously described^18^. In each experiment, half of the embryos were subjected to treatment while the other half was maintained in fishwater as a control. Treated and untreated embryos were kept in separate incubators to avoid any possible vapor-related contamination from evaporated chemicals. After 48 h of compound exposure, animals were tallied according to phenotype. Chi-square analyses were performed to evaluate differences between wildtype and edema phenotypes. Multivariate analysis was used to separate effects from morpholino exposure versus compound exposure. Statistical software used for analyzing the data was JMP version 14.0.

### Staining and imaging

Hemoglobin staining was performed as described previously^27^. Dechorionated live embryos at 72 hpf were stained in 0.6 mg/mL o-dianisidine (Sigma-Aldrich, St. Louis, MO) containing 0.01 mol/L sodium acetate (pH 4.5), 0.65% H_2_O_2_, and 40% ethanol in the dark for 30 min. Embryos were then dehydrated through graded ethanol washes of 50%, 75%, and 100%, for 5 min each. Finally, embryos were cleared in benzyl benzoate and benzyl alcohol at a 2:1 ratio. Embryos were stored in the clearing solution until imaged using a Leica DFC340FX fluorescent microscope with PlanAPO 1.6×/0.05 NA objective (Leica Camera Inc., Allendale, NJ, USA). Image capture was done with Leica LAS software, and post processing was done using Adobe Photoshop CS4 (Mountain View, CA, USA).

## Supplementary information

Supplementary Figure 1.

## Data Availability

The datasets generated during and/or analyzed during the current study are available from the corresponding author on reasonable request.
