# Supplementary material for: Traditional African remedies induce hemolysis in a glucose-6-phopshate dehydrogenase deficient zebrafish model
Source: Sci Rep. 2020 Nov 5;10:19172. doi: 10.1038/s41598-020-75823-x (PMC7645625; doi:10.1038/s41598-020-75823-x)
Supplement: Supplementary file 1 — Supplementary Figure 1. [file 41598_2020_75823_MOESM1_ESM.pdf]

Traditional African remedies induce hemolysis in a glucose-6-phopshate dehydrogenase deficient zebrafish model

Olufunmilayo Arogbokun MPH, Margaret Shevik MD, Tina Slusher MD, Zubaida Farouk, Alexis Elfstrum, Jenna Weber, Sarah E. Cusick PhD, Troy Lund MD, Phd

A

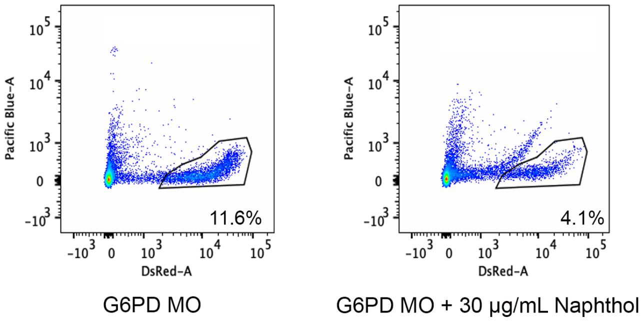

B

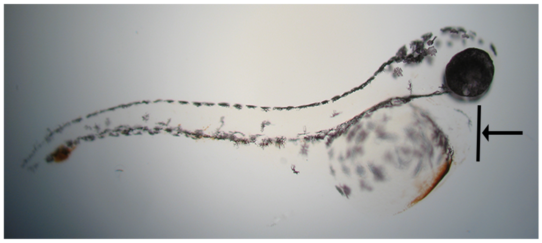

Supplemental Figure S1. 1-naphthol induced hemolysis in G6PD morphants. (A) *Gata1*:DsRed embryos are characterized to express DsRed in erythrocytes[1] and were injected with G6PD morpholinos, dechorionated, and exposed to 30 µg/mL 1-naphthol for 48 hours as per the Methods section[2]. Single cell suspensions were prepared and DsRed positive erythrocytes were enumerated by flow cytometry as previously reported[2]. (B) Micrograph shows an example of loss of erythrocytes and edema in a G6PD morphant exposed to 30 µg/mL of 1-naphthol. Animals were fixed and stained with o-dianisidine as previously reported[2,3]. The vertical bar and arrow indicate the extent of cardiac edema which is difficult to visualize under the given light/contrast settings.

1 Traver, D. *et al.* Transplantation and in vivo imaging of multilineage engraftment in zebrafish bloodless mutants. *Nature Immunology* **4**, 1238-1246, doi:10.1038/ni1007 (2003).

2 Patrinostro, X., Carter, M. L., Kramer, A. C. & Lund, T. C. A model of glucose-6-phosphate dehydrogenase deficiency in the zebrafish. *Exp Hematol* **41**, 697-710 e692, doi:10.1016/j.exphem.2013.04.002 (2013).

3 Detrich, H. W., 3rd *et al.* Intraembryonic hematopoietic cell migration during vertebrate development. *Proc Natl Acad Sci U S A* **92**, 10713-10717 (1995).
